# Breaking Bad News in Obstetrics and Gynecology: We Must Talk About It

**DOI:** 10.1055/s-0042-1742316

**Published:** 2022-07-12

**Authors:** Luísa Silva de Carvalho Ribeiro, Bárbara Flecha D'Abreu, Aline Evangelista Santiago, Eduardo Batista Cândido, Gustavo Salata Romão, Marcos Felipe Silva de Sá, Agnaldo Lopes da Silva Filho

**Affiliations:** 1 Department of Obstetrics and Gynecology , Faculdade de Medicina, Universidade Federal de Minas Gerais, Belo Horizonte, MG, Brazil; 2 Department of Tocogynecology, Universidade Estadual Paulista Júlio de Mesquita Filho, Botucatu, SP, Brazil; 3 Faculdade de Medicina, Universidade de Ribeirão Preto, Ribeirão Preto, SP, Brazil; 4 Department of Obstetrics and Gynecology, Faculdade de Medicina, Universidade de São Paulo, Ribeirão Preto, SP, Brazil

**Keywords:** communication, bad news, obstetrics, gynecology, medical education, comunicação, más notícias, obstetrícia, ginecologia, educação médica

## Abstract

Breaking bad news is common in obstetrics and gynecology (ob-gyn). However, it is difficult, and few doctors receive training on how to deal with this situation. This narrative review aims to gather, analyze, and synthesize part of the knowledge on the area, focused on Ob-Gyn. Among the 16 selected articles, two are randomized controlled intervention studies, and most studies refer to obstetrics. The results found by us pointed out that simulation, feedback/debriefing, lectures, and protocols could improve doctors' performance in communicating bad news. For patients, the context and how the information is transmitted seem to impact more than the content of the news. Ob-Gyn doctors could benefit from specific protocols and education, given the specialty's particularities. There is a lack of evidence about the most effective way to conduct such training. Finding validated ways to quantify and classify studies' results in the area, which would allow for the objective analysis of outcomes, is one of the biggest challenges concerning this topic.

## Resumo

Dar más notícias é comum em obstetrícia e ginecologia. Porém, é difícil e poucos médicos recebem treinamento sobre como lidar com essa situação. Esta revisão narrativa tem como objetivo reunir, analisar e sintetizar parte do conhecimento sobre a área, com foco na obstetrícia. Dentre os 16 artigos selecionados, dois são estudos de intervenção randomizados e controlados, e a maioria dos estudos refere-se à obstetrícia. Os resultados encontrados ressaltaram que simulação, feedback/entrevistas, palestras e protocolos podem melhorar o desempenho dos médicos na comunicação de más notícias. Para os pacientes, o contexto e como as informações são transmitidas parecem ter maior impacto do que o conteúdo das notícias. Os obstetras e ginecologistas poderiam se beneficiar de cursos e protocolos específicos, dadas as particularidades da especialidade. Faltam evidências sobre a forma mais eficaz de realizar esse treinamento. Encontrar formas validadas de quantificar e classificar os resultados dos estudos na área, permitindo uma análise objetiva dos resultados, é um dos maiores desafios neste tema.

## Introduction

“Bad news” is generally defined as any information that negatively impacts the life or vision of the future of those who receive it, according to their perspective. 1 2 Due to this subjectivity, diagnoses with different degrees of complexity may be considered bad news. In addition to the impact caused by the content of the news itself, how communication occurs directly influences patient's ability to face the diagnosis. This communication is also an important aspect of the doctor-patient relationship, and influences the patient's satisfaction with the service provided. 1 2 3 Therefore, the way doctor-patient communication occurs can be more harmful than the diagnosis itself. 4

Most physicians consider it an arduous task to transmit bad news, and the majority does not receive formal training for it in college, medical residency, or specialization programs. 1 4 5 It is believed that this ability is acquired in daily practice, throughout the individual's own practice, or by observing more experienced professionals. However, this is not corroborated by the literature, and the lack of adequate training is an important cause of the great stress that health professionals experience while transmitting bad news, which negatively impacts doctor-patient communication. 1 4 5 6 This stress may aggravate the occurrence of burnout syndrome among medical professionals. 3

The setting up, perception, invitation, knowledge, emotions, strategy and summary (SPIKES) protocol, created in the 1990s, aims to assist oncologists in breaking bad news by suggesting a strategy in the communication process. 2 5 This protocol is increasingly used and adapted for different areas, including obstetrics and gynecology (ob-gyn).

Specific literature on breaking bad news is scarce, although it has expanded in recent years. 5 Specifically in Ob-Gyn, the numbers are even smaller despite the recurrence of this type of communication in the area, which may involve pregnancy loss, fetal malformations, maternal complications during pregnancy, neoplasms, infertility, sexually transmitted infections, and any other diagnoses that negatively impact the patient's life. The present narrative review aims to gather, analyze, and synthesize part of the existing knowledge about breaking bad news in Ob-Gyn, which are specialties with many particularities.

## Methods

For this narrative review, we searched for publication in the databases PubMed, Scielo, Medline via Ovid, and Portal de Periódicos CAPES between April 2020 and February 2021, using the descriptors *breaking bad news obstetric* , *breaking bad news gynecology* , *breaking bad news* , *health communication* , *bad news obstetrics* , *bad news gynecology* , *SPIKES protocol obstetrics* , *SPIKES protocol gynecology* , *SPIKES protocol* and their Portuguese equivalents.

**Fig. 1:**
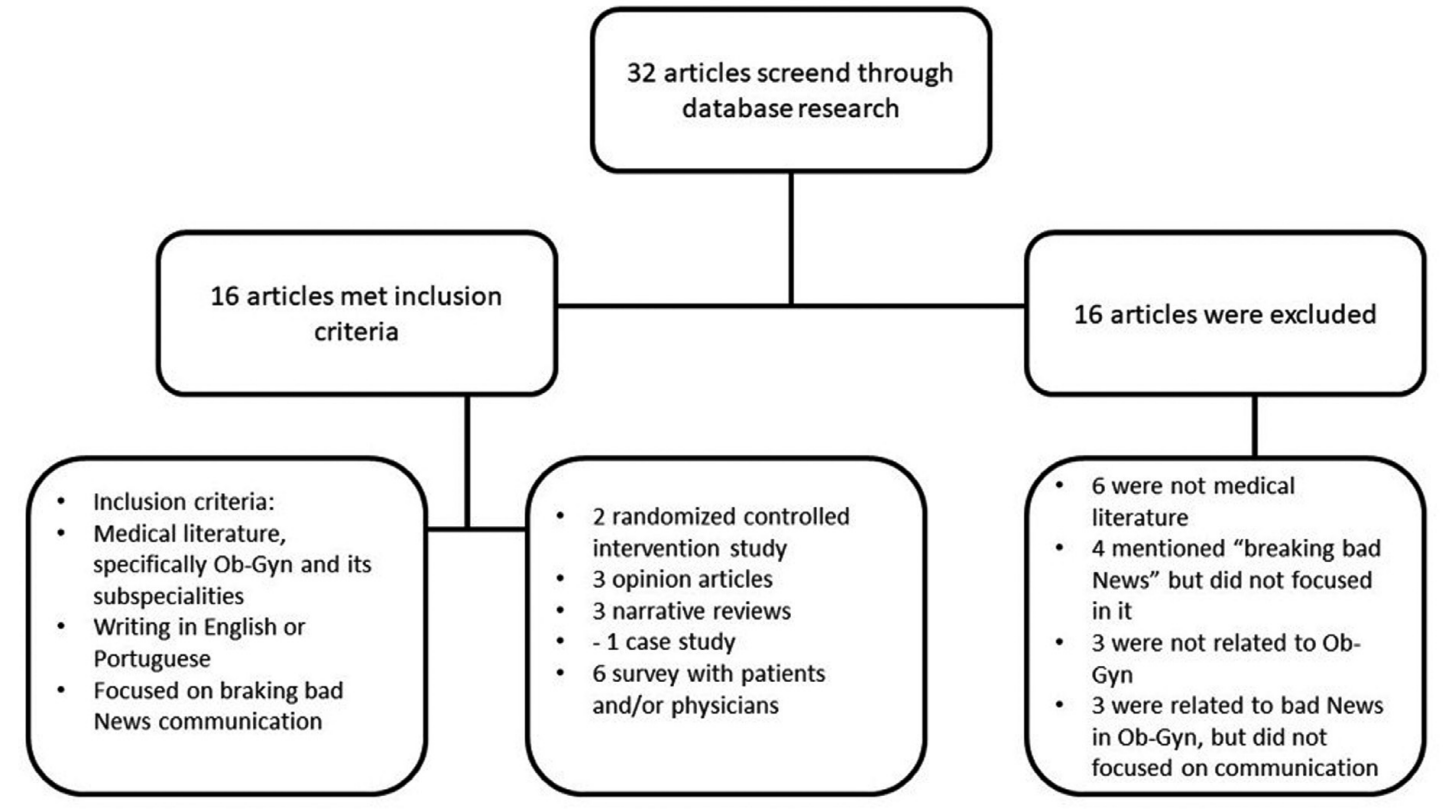
Flowchart: selection process for the article.

A total of 32 articles were selected and reviewed ( Fig. 1 ). Sixteen studies met the following inclusion criteria: belonging to the medical literature, specifically regarding ob-gyn and their subspecialties, being written in English or Portuguese, and focusing on breaking bad news. The exclusion criteria removed 16 articles, due to the following reasons: were related to ob-gyn but not in the medical field; mentioned “breaking bad news” but not as the focus of the study; were not associated with ob-gyn; were related to bad news in ob-gyn but did not focus on medical communication.

The 16 articles selected for this review are described in Table 1 . Two of them were randomized controlled intervention studies. Opinion articles, narrative reviews, surveys, and a case study form the other publications included. Most refer to obstetrics.

**Table 1:** Articles selected for the review

Author and year of publication	Area	Comments
Setubal et al. 2018 7	Obstetrics	Randomized controlled intervention study. Case simulation of perinatal loss: a resident physician should inform the occurrence to a simulated patient (the parent), followed by instant feedback from the actor, and both moments are filmed. The participants were then randomly allocated to the intervention group (video review + training using the SPIKES protocol) or the control group (without training). A similar simulation was then performed and analyzed, with the actor blind to the intervention.
Karkowsky et al. (2016) 8	Obstetrics	Randomized controlled intervention study. Doctors underwent simulation of reporting a fetal death and care plan for the case, followed by immediate performance evaluation from the actor, the examiner, and themselves. After, they were divided randomly into two intervention groups: 1) a lecture about breaking bad news; 2) a debriefing session with the examiner. A new simulation was then performed, with different actors and examiners, blind to the intervention type, followed by further analysis. Six months after training, the researchers would contact the participants to assess the level of knowledge retention.
Guerra et al. (2011) 9	Fetal medicine	Opinion article
Lim et al. (2011) 10	Obstetrics	Opinion article
Nuzum et al. (2017) 11	Obstetrics	An in-depth qualitative study based on interviews with grieving parents about their memories and insights regarding the moment they received a fetal death diagnosis.
Greiner and Conklin (2015) 12	Obstetrics ultrasonography	Narrative review
Romm (2002) 13	Gynecology and obstetrics	This article presents doctors' and patients' perceptions about the impact in communication skills produced by lectures given by patients to doctors, reporting their experiences in receiving bad news.
Leone et al. (2017) 14	Reproductive medicine	Doctors formed discussion groups to share their own experiences in communicating bad news and discuss the SPIKES protocol's applicability in the reproductive medicine area.
Lalos (1999) 15	Reproductive medicine	Opinion article
Kuroki et al. (2013) 16	Gynecologic oncology	Survey about patients' satisfaction regarding several aspects presented or not when they received bad news.
Zanetti-Dällenbach et al. (2006) 17	Mastology/ Obstetrics	Case study
Alkazaleh et al. (2003) 18	Obstetrics ultrasonography	Survey about patients' perception of the moment they received bad news and their preferences for such a situation.
Cockburn and Walters (1999) 19	Gynecology and obstetrics	Narrative review
Karkowsky and Chazotte (2013) 20	Gynecology and obstetrics	Narrative review
Setubal et al. (2017) 21	Obstetrics	Physicians' (participants) perception of the training on communicating bad news reported by Setubal et al. (2018). 7
Johnson et al. (2019) 22	Obstetrics ultrasonography	Analysis of a questionnaire applied to sonographers about training in breaking bad news, burnout syndrome, and psychological well-being.

## Results/Discussion

### Specific aspects of Gynecology and Obstetrics

Breaking bad news in the context of ob-gyn has some particularities. The occurrence of adverse events during pregnancy or childbirth destroys the expectation and frustrates plans made about the supposedly healthy child to be born. 7 8 9 10 11 Pregnancy losses are considered one of the most stressful events in adulthood, and can trigger grief reactions. Mothers who experience fetal death are more likely to experience complicated and prolonged grief, which can last for more than a year. 7 10 Feelings of guilt, anxiety, eating disorders, and shame are additional reactions experienced by parents who suffer pregnancy losses. 9 10 From the perspective of health professionals, communicating fetal death is one of the most difficult duties, especially at the beginning of their careers. 7 11

When receiving bad news, the patient's anxiety is greater when she does not expect something adverse to happen. 9 This happens, for example, during ultrasound exams, as some women ignore its real purposes and consider it to be only a source of entertainment, a moment of personification of the fetus, and materialization of pregnancy expectations. 9 12 The literature suggests patient education about ultrasound purposes, both by the requesting physician and by the examiner, as an attempt to avoid this situation. 9 It is also important that the sonographer informs, when suitable, that they will perform the exam in silence and then discuss the findings with the patient, because unexpected silence may increase anxiety since the patient might think that something wrong with the test, even if it is not. 9 12 13

Additionally, regarding obstetrics—unlike other areas—the patient is no longer a single individual, but becomes the mother-fetus binomial and, when present, the partner is also considered as part of the “patient.” Thus, both parents should be present when the bad news is given for the first time, so that the mourning can start simultaneously. It is important that the doctor be aware that parents usually have different reactions when receiving bad news and it is their duty to understand and comfort both. 7 12

This perspective of the patient as more than one individual is also observed in reproductive medicine, where the couple is the patient, which can make it even more difficult to transmit negative news. 14 15 Receiving an infertility diagnosis brings patients the feeling of frustration of an entire life plan, and has negative impacts on self-esteem, sexuality, and the couple's relationship. It also creates feelings of shame and guilt. Leone et al. 14 further claim that receiving a diagnosis of infertility may have the same psychological burden as receiving a breast cancer diagnosis. Also, in human reproduction, bad news may occur repeatedly and might have to be transmitted several times, with each treatment failure, for example. 14 15

When concerning gynecological cancer diagnosis, most of the time, it is announced by physicians who are not specialists in oncology, reinforcing the importance of improving communication skills for medical professionals of all areas. 16 Another rare and specific situation in ob-gyn is the communication of cancer diagnosis during a pregnancy, because situations like this contrast the extremes of life: possible terminality *versus* the development of a new infant. The approach of these patients requires a multidisciplinary team, and communication must be done by a trained, preferably more experienced, professional. 17

### What are the Patients' Perspectives?

The doctor's lack of preparation to transmit bad news affects the patient. According to studies, the negative memories they have of the moment they received bad news are related to the report's content itself and the doctor's lack of skills for communication. 7 9 11 How the information is transmitted is one factor that influences how traumatic this event will be for the patient. 18 Patient satisfaction with medical care is based mainly on the doctor-patient relationship, which is greatly influenced by the physician's communication skills. 19 Dissatisfaction with this topic is an important cause of complaints and lawsuits against doctors and health care institutions. 14 19 20 In line with this, Kuroki et al. 16 and Alkazaleh et al. 18 showed that the patient's degree of satisfaction with communicating bad news was not completely related to the severity of the malignant neoplasm or fetal malformations, respectively, but to the physicians' attitudes. 16 18

When receiving bad news, patients may experience the grief reactions: denial, anger, negotiation/bargaining, depression, and acceptance. 9 12 15 These emotions, added to anxiety, tension, and the amount of new information provided, can compromise the patient's assimilation of the content, which would cause them to forget up to two-thirds of the information given. 9 19 Therefore, physicians should communicate bad news while respecting the patient's time to assimilate it, and repeat information if necessary. 9 The use of excessively technical language also impairs assimilation and should be avoided. 9 19

Strategies to improve the patient's understanding of their health status include scheduling return visits, reviewing the content at the end of the appointment, offering help to inform anyone the patient wishes, and providing written materials containing information about the diagnosis. 9 12 17 19 The presence of a companion also helps with the transmission of information, as having another individual to hear and remember the news, can give the patient time to process their emotions, and comfort them. 9

Of the articles chosen for our analysis, 4 focused on patients' perception of the moment they received bad news from the attending physician ( Table 1 ). Kuroki et al. 16 showed, with statistical significance, that the patients were more satisfied when they received the information in person during meetings lasting more than 10 minutes, set in a private, quiet, and comfortable environment, free of interruptions. When communication was patient-centered—that is, when patients were able to ask questions, and the doctor took into consideration the patients' needs and previous knowledge about their clinical condition—the satisfaction was higher. 16

The other factors related to medical behavior that also positively impacted patient satisfaction were: being contacted later by the doctor for new explanations, sensing that the doctor is not nervous when giving the information, recognizing correct use of body language by the doctor. 16 Adding to that, follow-up medical visits was also identified by Leone et al. 14 as a factor that increases patient satisfaction. The adequate use of body language is reinforced by Guerra et al. 9 and Greiner and Conklin. 12 How much the patient trusts the physician also interferes with satisfaction levels. 16

In the study by Romm, 13 patients also point out the aspects identified by Kuroki et al., 16 listed above, as crucial for proper communication of bad news, and added: providing information also in writing; allowing the presence of a companion, if it is the patient's wish; and passing the news as soon as possible, as long as it is not by text message or while the patient is at work. 13 16 Regarding the physician's demonstration of empathy, patients dislike expressions like: “I know what you are going through” or “I know how you feel,” unless the professional has experienced a similar situation themselves. 13 The last point is reinforced in the review by Greiner and Conklin. 12 However, hearing from doctors that they are “very sorry for what the patient is going through” was considered desirable by the participants. Finally, patients pointed out that it was fundamental that the physician delivered the news entirely and in detail without ending the patient's hopes. For this, they suggest doctors do not provide statistical data unless they are requested to do so. 13 Cockburn and Walters 19 reinforced the importance of maintaining the patient's hopes and advise doctors to encourage them to express themselves emotionally and to ask questions.

Alkazaleh et al. 18 applied questionnaires to women who received the diagnoses of fetal malformations during an obstetric ultrasound. The results showed that patients consider the quality of the information the most important aspect when communicating bad news; this includes clarity of language, time to ask questions, receiving written information, and data about the care plan. In agreement with the previously mentioned studies, other points considered as positive were: how quickly they are informed about the diagnosis; the opportunity to have a companion; patient-centered communication; doctors' empathy; and a private environment when receiving the news. 18 This last aspect is also reinforced by Cockburn and Walters. 19

In agreement with Kuroki et al., 16 who showed no relationship between the specific aspects of the tumor and the degree of satisfaction with care, Alkazaleh et al. 18 reported that the severity of the fetal malformation diagnosed did not impact the patient's satisfaction. 16 18

Nuzum et al. 11 collected data from interviews with grieving parents about their perception of the moment they received fetal death diagnosis. They reinforce the importance of adequate language used, the sensitivity from the doctor, and proper environment. Additionally, physicians' use of “diversionary techniques”—for example, omitting the news while waiting for a second opinion—was considered a negative attitude during communication.

The literature brings other points that improve transmission of bad news. For example, in the context of obstetric ultrasound, Guerra et al. 9 recommends for the patient to be dressed, sitting at the same height and facing the doctor; eye contact is of great importance when receiving the news. Besides, in case of severe ultrasound findings, it is advisable to leave the patient and her companion alone in the room for some time after breaking the news, so they are free to express themselves. 9

### How to Improve Medical Training?

Among the reviewed articles, several reinforce the impact of the doctors' lack of preparation for communicating bad news through academic and professional life. 7 9 19 20 Setubal et al. 21 highlight the deficiency of this type of training in ob-gyn residences. Negative feelings experienced by physicians while communicating bad news make the task even more complicated, and excessive stress during this moment can be a risk factor for burnout syndrome among medical professionals. 7 8 20 21 22 It is mistakenly believed that the practice and observation of more experienced colleagues breaking bad news could provide adequate training for this situation, but this training method allows for reproduction and perpetuation of errors. 7 12 19 21

Appropriate strategies for communicating bad news can be taught, just like other medical skills, and many doctors recognize the importance of such training. 8 19 20 21 Adequate preparation can prevent emotional damage for both doctors and patients. 13 21 There is a lack of evidence in the literature about the most effective way to teach communication of bad news and how to keep this training up to date. 16 20

Setubal et al. 7 showed that simulation activities followed by immediate feedback—and, in the intervention group, also a lecture on the SPIKES protocol—were able to increase ob-gyn and pediatrics residents' performance in breaking bad news. The benefit of the simulation followed by feedback or debriefing is also recognized by Karkowsky et al. 8 and Leone et al. 14 However, this method may require financial and human resources not available in all institutions. 21

When assessing the perception of resident physicians participating in the study, most were satisfied with the methods applied and would recommend the training to a colleague. 7 Almost all of the participants noticed an improvement in their skills and knowledge on how to deliver bad news, and believed that training would improve their practice. There was a consensus on recognizing the SPIKES protocol's great value to systematize bad news communication in clinical practice. 21

In a similar randomized controlled study, Karkowsky et al. 8 showed that the self-perception of ob-gyn doctors about their performance in communicating bad news improved after a simulated situation of breaking bad news, followed by a lecture on the topic or an individual debriefing session with a specialist, and then a new simulation. The individual perception of improvement was greater in the debriefing group, as the individuals who underwent debriefing appeared to have slightly greater improvement in verbal and non-verbal communication skills. Finally, when analyzing the ability to retain the knowledge learned after 6 months of the intervention, the results were satisfactory in both groups. Romm 13 also used a lecture as an attempt to enhance doctors' communication abilities and participants reported an improvement in recognizing key points when transmitting bad news.

In contradiction to the majority of the other articles, a survey performed by Johnson et al., 22 with sonographers from the United Kingdom, showed that the majority had received some training for communicating bad news. Lectures and discussion groups were the most common teaching methods. However, the preferred learning methods chosen by the professionals were observation of clinical practice and patient feedback. There was no correlation between receiving training on breaking bad news and the professional's psychological well-being or the occurrence of burnout syndrome. Still, training was associated with lower occurrence of loss of motivation toward the job. 22

Finally, Leone et al. 14 concluded that the SPIKES protocol might benefit reproductive medicine doctors by reducing stress associated with communicating bad news. This protocol is also useful in other subspecialties of ob-gyn, as doctors consider dealing with the patient's emotions one of the most challenging steps. 7 10 12 19

In Brazil, communication skills and the ability of communicating bad news are part of the Professionalism Axis of the Competency Framework in Gynecology and Obstetrics, which was approved by the National Commission for Medical Residency (CNRM-MEC) as the official reference for training in this specialty. Therefore, all medical residency programs must offer the necessary training in these skills, as well as evaluate their acquisition by resident physicians. 23

Communicating negative news is part of the routine of Ob-Gyn. However, formal training for this skill is not yet part of several residency programs and is equally deficient in medical school. 5 7 8 11 There is a belief that doctors learn how to break bad news in daily practice, or by observing more experienced doctors perform such a task. However, the literature shows no difference in this ability when comparing medical students, residents, and specialists. 1 5 6 7 11 19 21 Additionally, learning by observation favors the perpetuation of errors. 7 This highlights the importance of implementing formal training in communicating bad news in medical education, given the negative impacts caused by poor communication. The principle of primum non nocere should not be forgotten. 9 Although physicians cannot modify the content of negative news, they can improve their communication skills. 11 Unfortunately, there is still no evidence of the most effective way to conduct such training. 1

Initially designed for oncology, the SPIKES protocol has been used in several medical specialties, as well as in medical school, for training medical professionals on how to break bad news to patients. 2 3 5 20 The strategies proposed by this protocol seem to be suitable for ob-gyn, resulting in the improvement of the professionals' performance and a greater personal satisfaction while communicating bad news to patients. 7 10 11 14 21 However, ob-gyn has several particularities, and may benefit from specific protocols. 8 20 In obstetrics and ultrasound, for example, the diagnosis and communication occur almost simultaneously, and there is no time to prepare the environment or for doctors to prepare themselves, which would be the first stage of the SPIKES protocol. 9 20 Another particular aspect of the ob-gyn specialty is that the patient is often a couple, requiring different approaches for each of the individuals involved. 9 14 15 The key points of the present review are collected on Table 2 .

**Table 2:** DOs and DON'Ts in communicating bad news in gynecology and obstetrics

Breaking bad news in Ob-gyn
**DOs**
Follow the SPIKES protocol, adapting it to ob-gyn reality.
Study and practice “communicating bad news” as any other medical professional should.
Try to guarantee the patient's privacy and an environment free of interruptions when breaking bad news.
The communication must be patient-centered.
Express empathy and hope.
Review the information with the patient and offer written material about it.
In obstetrics, consider both the parents and the fetus as the patient. Work with a multidisciplinary team whenever possible.
**DON'Ts**
Avoid expressions like “I know what you are going through” or “I know how you feel,” unless you have gone through a similar situation.
Avoid excessively technical language.
Do not provide statical data unless you are requested to do so.
Do not omit, minimize, or delay the information, if the patient desires to hear it.
Do not communicate bad news through impersonal means, such as voice/text messages.

Abbreviation: Ob-gyn, obstetrics and gynecology.

While protocols and training about how to break bad news can improve the physician's ability and decrease anxiety, this will never be never easy. It is an unwanted situation, both for doctors and patients, and it is made harder by the unpredictability of the patients' reactions to highly stressful situations. 14 21

Despite analyzing only part of the existing studies on the subject, this review shows that the communication of bad news is very recurrent and particular in ob-gyn, and the lack of preparation for such a situation is harmful to doctors and patients. It might be hard to produce robust evidence on the matter because of the absence of objective results to be analyzed, as the results achieved with improving communication are mostly subjective. Undoubtedly, finding validated instruments to quantify and classify such findings to allow a statistical and objective analysis of outcomes is one of the biggest challenges in the area.
